# Targeted Interference with USF2 Binding to the SERPINE1 Proximal Promoter E-Box in Dual Mutant p53^R282Q,H179Y^ Human Keratinocytes Inhibits Serum-/TGF-β1-Induced SERPINE1 Expression and Stimulates Epithelial Cell Proliferation

**DOI:** 10.3390/biomedicines14030726

**Published:** 2026-03-22

**Authors:** Stephen P. Higgins, Ralf-Peter Czekay, Craig E. Higgins, Paul J. Higgins

**Affiliations:** Department of Regenerative and Cancer Cell Biology, Albany Medical College, Albany, NY 12208, USA; higgins@amc.edu (S.P.H.); higginc@amc.edu (C.E.H.)

**Keywords:** USF1, USF2, E box, SERPINE1, p53, HaCaT cells human keratinocytes, cell cycle, transcription, growth control, decoy

## Abstract

The SERPINE1 gene encodes the serine protease inhibitor plasminogen activator inhibitor type-1 (PAI-1), a major negative regulator of the plasmin-dependent pericellular proteolytic cascade and a crucial determinant in the program of stromal remodeling. Recent omics approaches confirmed that high tumor SERPINE1 levels are prognostic for poor disease outcomes and shorter disease-free survival in various malignancies. Kinetic analysis of biomarkers of cell cycle transit in growth-synchronized p53 dual mutant human keratinocytes confirmed that PAI-1 transcription occurred early after growth activation of quiescent (G_0_) cells and prior to G1 entry. Previous evidence has confirmed that differential residence of USF family members (USF1→USF2 switch) at the PE2 region hexanucleotide E box motif (CACGTG) in the SERPINE1 proximal promoter characterizes the G_0_→G_1_ transition period and the transcriptional status of the SERPINE1 gene. A consensus PE2 E box motif (5′-CACGTG-3′) at nucleotides −566 to −561 is required for USF occupancy of the PE2 E box and serum-stimulated SERPINE1 transcription. Interference with USF2 occupancy of the PE2 E Box site by a double-stranded PE2 “decoy”, or induced expression of a dominant-negative USF (A-USF) construct, attenuate serum- and TGF-β1-stimulated SERPINE1 synthesis. Tet-Off activation of an A-USF insert reduced both PAI-1 and PAI-2 transcripts while increasing the fraction of proliferating (Ki-67^+^ cells). Conversely, overexpression of USF2 or adenoviral delivery of a PAI-1 vector inhibited HaCaT colony expansion. These findings are discussed in this review and collectively suggest that the USF1→USF2 transition at the PE2 E box site and subsequent SERPINE1 transcription impact serum-stimulated keratinocyte growth and, likely, cell cycle progression.

## 1. Introduction

Upstream stimulatory factor-1 and -2 (USF1/2) are members of the conserved basic helix-loop-helix/leucine zipper (bHLH-LZ) MYC family of transcription factors [1,2]. USF1/2 bind to core E box sequences (CANNTG) as tetramers in the promoter regions of target genes which encode proteins that regulate various basic processes, including development, cellular growth and proliferation, the immune response, metabolic pathways, and cancer progression [1,2,3]. Multiple signaling networks and their activated protein kinases influence USF1/2 function largely through site-specific phosphorylation (primarily on serine and threonine residues) while USF1/USF2 homo- and heterodimer composition and recruited co-factors dictate transcriptional controls on USF responsive genes [4,5,6,7]. Earlier work indicated that both USF1 and 2 inhibit myc oncogene-mediated cell transformation; however, USF2 has more widespread anti-proliferative properties and attenuates tumor cell focus-formation in response to E1A + ras and mutant p53 + ras oncogenes [8]. Perhaps not unexpectedly, therefore, Crispr-Cas9-directed elimination of USF2 in mouse embryonic fibroblasts promotes both cellular growth and migration [9]. Conversely, USF1/2 overexpression suppressed proliferation of normal and malignant thyrocytes, as well as other cell types, by delaying G_2_/M transit, likely as a consequence of down-regulation of cyclin B1 and CDK1, c-MYC and induction of p27 and p53 [7,10,11]. It is now apparent, however, that the cell cycle-related anti- vs. pro-proliferative functions of USF1/2 are context- and cell type-dependent and involve specific mitogen activated (p38, PKC, PKA) and cyclin-dependent (CDK1, CDK5) kinases as well as CK2, DNA-PK and GSK3β [6,7,8,9,12,13,14]. The generation of splice variants of USF1/2 also impacts transcriptional outcomes. The USF1 and USF2 genes, located on 1q22-q23 and 19q23, respectively, possess 10 protein encoding exons. Alternate transcript splicing involving exon 4 of USF2 generates the two isoforms USF2a (44 kDa) and USF2b (38 kDa), of which USF2b (lacking exon 4 sequences) functions as a dominant negative regulator of USF1 or USF2a target gene expression reviewed in [7].

Transcriptome analyses indicated that USF1/2 bind to E boxes in the promoter regions of over 2500 genes [15]. While loss of USF1/2 activity is evident in several tumor cell types, recent findings suggest that USFs may also be pro-tumorigenic [16,17] (reviewed in [18]). Omics approaches have illuminated the involvement of both USF1 and USF2 in several of the more lethal cancers, although the underlying mechanisms may vary or be tumor-type unique. USF1/2 abundance and, therefore, their transcriptional impact on individual responsive genes, appear critical in determining the tumor suppressive vs. promoting effects of USF family members [19,20,21]. In lung adenocarcinoma, for example, USF1 upregulated expression of the coiled-coil-helix-coiled-coil-helix domain containing 4 protein (CHCHD4) [22]. CHCHD4 is highly expressed in human malignancies where it is associated with disease progression, poor patient outcomes and increased recurrence. USF2 is also strongly expressed in the dysplastic pulmonary epithelium, in small cell lung cancer and squamous cell carcinoma [23]. The USF1 target genes lncRNA-NEAT1, TF-ETV5 and ZNF641 are similarly implicated in the progression of hepatocellular carcinoma [15,24], and USF1 stimulates growth and proliferation in breast cancer and glioblastoma by inducing expression of SEZ6L2 and the stemness protein CD90, respectively [25,26]. The LncRNA TUG1 recruits USF1 to increase transcription of ROMO1 and elevated ROMO1 expression induces an epithelial-to-mesenchymal transition (EMT) in hepatocellular carcinoma cells with increases in proliferation, migration and tissue invasion [27]. Elevated USF2 expression stimulates abnormal lipid metabolism in lung adenocarcinoma by transcriptionally activating PEX3 to upregulate and promote interactions with SLC25A17 [28]. USF2 knockdown inhibits abnormal lipid accumulation and attenuates tumor growth in nude mice while USF1 overexpression increases colony formation, survival and radio-resistance in prostate cancer cells [29]. USF2 induced transcription of the translational regulatory protein BZW2 facilitates colorectal cell proliferation, invasion, stemness and inhibition of apoptosis through a LAMP3-dependent mechanism [30].

At least some of these programmed changes appear due to a cooperative USF→TGF-β1 axis since both USF1 and USF2 stimulate TGF-β1 promoter activity and TGF-β1 protein secretion in specific cell types [10,31]. USF1 overexpression promotes both EMT and migration in melanoma cells by increasing TGF-β1 expression while USF1 knockdown attenuated both the morphologic transition and the motile response [32]. TGF-β1 may also function as an initiator, inducing USF2 to transcriptionally upregulate S100A8 in colorectal cells; S100A8, in turn, stimulates cell migration, invasion and EMT [33]. One interesting finding implicates USF2 as an inhibitor of Smurf1 and Smurf2 transcription [34]. Since Smurfs suppress TGF-β signaling, TGF-β reporter activity is significantly reduced by USF2 siRNA. USF2 promotes human breast cancer cell proliferation, invasion and migration [34]; it is tempting to speculate, therefore, that this may relate to USF2 activation of the TGF-β1 pathway leading to EMT and tumor aggressive behavior.

## 2. SERPINE1 (PAI-1) Is a Poor Prognosis Biomarker and Effector of Tumor Aggressiveness

The clade E member 1 serine protease inhibitor SERPINE1, also known as plasminogen activator inhibitor-1 (PAI-1), is a potent negative regulator of the pericellular proteolytic cascade and a key factor in the spatial/temporal program of stromal remodeling [35,36,37]. PAI-1 is also a prominent member of the serum-induced “wound-response” transcriptome [38,39]. In dual mutant p53^R282Q,H179Y^ immortalized keratinocytes (HaCaT cells), PAI-1 is required for TGF-β1-stimulated planar locomotion and invasion through Matrigel barriers, responses likely involving LRP1-dependent engagement of the Jak/Stat pathway [40,41,42]. Matricellular (i.e., extracellular matrix-anchored) PAI-1 also promotes acquisition of a mesenchymal-to-amoeboid phenotypic transition that correlates with activation of signaling networks and downstream target genes required for efficient 3-D “tissue” invasion [43]. The consistent association of PAI-1 expression with stromal restructuring [38,44,45] suggests that this SERPIN integrates cycles of cell-to-matrix adhesion/dis-adhesion with “scaffold” remodeling to initiate and maintain effective cellular migration [46]. The specific phenotypic impact of PAI-1, however, is significantly more complex and likely requires a balance between its intracellular and extracellular activities that may be both cell type- and stimulus-specific [47]. Indeed, several SERPINS (SERPINE1 [PAI-1], SERPINB1, SERPINB2) are prominent members of the stromal remodeling transcriptome and proteome [38,39]. PAI-1, most particularly, has several functions in the integrated control of focalized matrix restructuring, cell-to-substrate adhesion/detachment, migration and proliferation [44,45,46,48,49,50,51,52,53]. PAI-1 effectively limits plasmin generation within the pericellular space to maintain a supporting “scaffold” for cell movement [46] while also regulating urokinase plasminogen activator-dependent growth factor activation, thereby attenuating the associated proliferative response [54]. Concerning the latter, certain USF-target “growth arrest-associated” genes (i.e., p16^INK4a^, PAI-1) may synchronize the proliferative and motile phases of the adaptive and maladaptive tissue repair programs by inhibiting cell proliferation while promoting migration [54,55].

PAI-1 is also a major downstream TGF-β1 target gene; it is apparent that both PAI-1 and TGF-β1 promote tumor cell migration, amoeboid motility, and tissue invasion [56,57,58]. TGF-β1-induced cell movement is effectively attenuated by the small molecule PAI-1 inhibitor Tiplaxtinin, suggesting that the PAI-1 anti-proteolytic function is required for TGF-β1-stimulated locomotion. This is consistent with the recent finding that deficiencies in PAI-1 or TGF-β1 reduce cell motility [41]. Expression of a PAI-1-GFP fusion protein under the inducible control of +800 bp of the injury-activated PAI-1 promoter prominently “marks” keratinocyte migration trails. The use of PAI-1-null cells, knockdown approaches, PAI-1 add-back rescue, and neutralizing antibodies confirms the requirement for PAI-1 in cell movement [41]. Similarly, adipose mesenchymal stem cells stimulate cellular motility by modulating SERPINE1 expression in dermal fibroblasts and keratinocytes [59]. In glioblastoma cells, PAI-1 increased EMT characteristics, tumor cell invasion, and migration under hypoxic culture conditions, and these effects were mitigated by PAI-1 knockdown [60].

**Figure 1:**
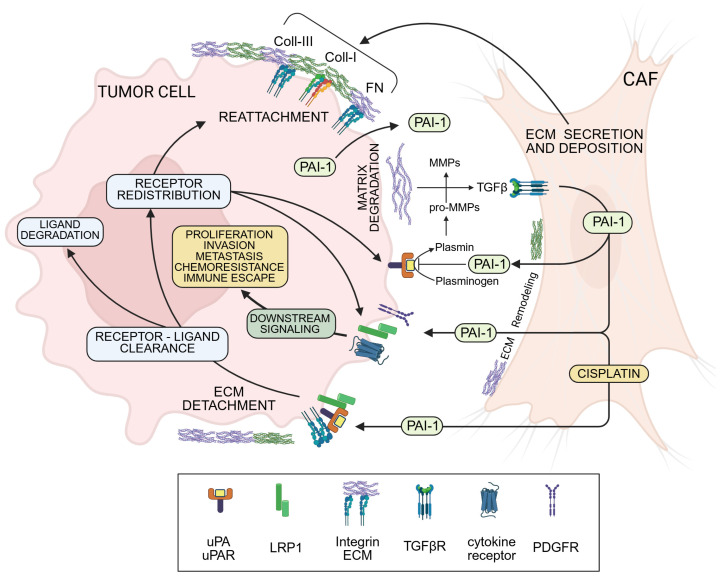
PAI-1 supports the emergence of a tumor aggressive phenotype by regulating multiple pathways in both tumor cells and stromal elements that coexist in the TME. Through interactions with the cell surface receptors uPA/uPAR and LRP1, PAI-1 drives a complex and multipronged program of tumor progression that impacts the spatial/temporal extent of matrix remodeling, promotes tumor cell survival, migration, tissue invasion, and acquisition of chemoresistance. Highly aggressive desmoplastic tumors are characterized by a well-developed fibrotic microenvironment largely provided by stromal CAFs that secrete extracellular matrix (ECM) proteins (Coll-I, Coll-III, and FN). uPA and MMPs promote the activation of matrix-bound latent TGF-β that further stimulates CAFs to express and release PAI-1 in the TME. Synthesis of PAI-1 by CAFs enables malignant cells to undergo an epithelial-to-mesenchymal transition (EMT) while upregulating tumor prosurvival anti-apoptotic pathways. PAI-1 interacts with and inhibits active proteinases (uPA) on the tumor cell surface, effectively reducing the pericellular proteolytic activity in the TME by blocking plasmin generation and subsequent pro-MMP activation. PAI-1 also deactivates cell adhesion receptors (integrins), resulting in local detachment of tumor cells from the stroma, initiating endocytic clearance (via LRP1) of proteinase receptors (uPAR) and integrins and, thereby, promoting redistribution and relocation of the cellular attachment and proteolytic apparatus to the leading edge of migrating and invading tumor cells rendering tumors more aggressive. The direct engagement of PAI-1 with LRP1 on tumor cells initiates collaborative cross-signaling with cytokine and growth factor receptors while activating potent pro-survival pathways. PAI-1-mediated cross talk between CAFs and cancer cells in the TME leads to multidrug resistance and metastatic spread. Chemotherapy (e.g., Cisplatin) also induces PAI-1 production in CAFs, thereby initiating mechanisms protecting neoplastic cels from such drugs. LRP1, LDL-receptor-related protein 1; MMP, matrix metalloproteinase; PAI-1, plasminogen activator inhibitor-1; TME, tumor microenvironment; uPA, urokinase-type PA; uPAR, uPA receptor. Figure 1 created in BioRender. Czekay, R. (2026). https://BioRender.com/y4ykca3 (accessed on 20 February 2026).

High tumor SERPINE1 levels predict poor outcomes and shorter disease-free survival in various human cancers [58,61,62,63,64,65]. SERPINE1 is a prominent member of the validated five-member EMT-related prognostic gene set in gastric carcinoma and the six-gene signature that is prognostic for reduced survival in head and neck squamous cell carcinoma patients, as well as indicative of a poor prognosis and higher risk score. SERPINE1 is a clinically important hub gene in a wide spectrum of human malignancies, and a strong indicator of reduced patient survival [66,67,68,69,70,71,72]. While many cell types in the tumor microenvironment (TME) synthesize and secrete PAI-1, cancer-associated fibroblasts (CAFs) are a particularly rich source of this SERPIN (Figure 1). PAI-1 co-localizes, in fact, to α-SMA-positive fibroblastoid cells at the tumor invasive margins and stroma-enriched regions [73,74,75,76,77,78,79]. The myofibroblastoid CAF phenotype (α-SMA^high^/PAI-1^high^), moreover, is involved in the development of tumor chemotherapeutic resistance and/or acquisition of a more aggressive, invasive phenotype [80,81]. Functional blockade of PAI-1 activity (e.g., with small molecule inhibitors) may be one approach to enhance chemotherapeutic efficacy [81].

## 3. Kinetics of Induced SERPINE1 Transcription in Dual Mutant p53^R282Q,H179Y^ Human Keratinocytes

**Figure 2:**
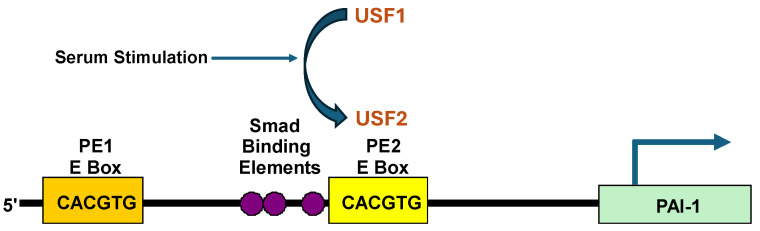
Position of two E boxes in the PAI-1 promoter. PAI-1 transcription in response to addition of serum to G_0_ cells involves a USF subtype switch (USF1→USF2) at the PE2 site E box motifs (5′-CACGTG-3′) in the PF1 region (nucleotides −794 to −532) of the PAI-1 promoter. Although chromatin immunoprecipitation analysis indicated a USF1→USF2 replacement at both E boxes, luciferase reporter gene assessments indicated that the PE2 E Box motif, which is 3′ downstream of three Smad binding elements, was more significant, at least for TGF-β1-induced PAI-1 promoter activation [5,84,85].

The SERPINE1 gene is a major TGF-β1 and p53 target and is robustly upregulated in response to serum-stimulation of growth-arrested G_0_ (quiescent) cells [5,40,54,82,83,84,85]. Kinetic modeling using established biomarkers of cell cycle transit (c-MYC; cyclin D_1_; cyclin A) in synchronized human (HaCaT) keratinocytes confirmed that PAI-1 transcription occurs early (prior to G_1_ entry) after serum-stimulation of quiescent (G_0_) keratinocytes [5,39,85]. Induced PAI-1 expression involves a USF1→USF2 subtype switch at the PE2 site E box motif (5′-CACGTG-3′) in the PF1 region (nucleotides −794 to −532) of the PAI-1 promoter [5] that resides three nucleotides downstream of a trio of TGF-β-responsive SMAD-binding elements (ACAG motif) at nucleotides −569 upstream of the transcription start site [85]. (Figure 2). Chromatin immunoprecipitation confirmed that the growth factor-responsive PE2 E box sites in the PAI-1 gene are, indeed, USF target sequences in vivo and that serum-stimulated PAI-1 expression reflected a dynamic USF1 → USF2 switch at this CACGTG motif [5]. A consensus PE2 E box motif (5′-CACGTG-3′) at nucleotides −565 to −560, moreover, is required for USF E box occupancy and serum-stimulated PAI-1 transcription. Indeed, a CG→AT substitution at the two central nucleotides ablated binding of USF to a mutant probe target and inhibits expression of a PAI-1 promoter-driven reporter [5], establishing the importance of an intact PE2 E box consensus sequence in serum-stimulated PAI-1 transcription.

PAI-1 mRNA levels rapidly decline several hours prior to the onset of S phase [5,55], suggesting that PAI-1 may negatively impact cell growth. PAI-1 knockdown in fact results in escape from senescence-associated proliferative arrest and TGF-β1-induced cytostasis in several cell types, including HaCaT keratinocytes [54,83]. The USF1 → USF2 transition at the PAI-1 PE2 E box, and subsequent PAI-1 transcription may regulate the time course of tissue remodeling progression through the cell cycle as part of the proliferative control program.

## 4. Interference with USF-DNA Binding Inhibits PAI-1 Expression and Stimulates Epithelial Cell Proliferation

**Figure 3:**
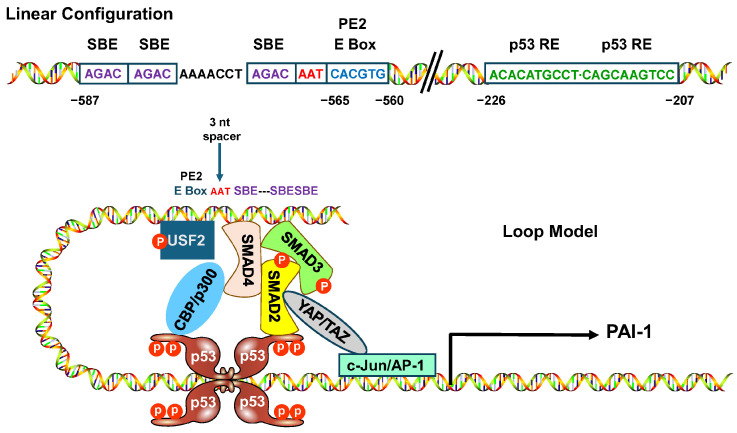
Construction of a transcriptional competent complex on the PAI-1 promoter. The linear configuration of the PAI-1 proximal promoter illustrates the spatial distribution of SMAD-binding elements (SBE), the 3 nucleotide AAT spacer, and the two p53 response elements relative to the PE2 E Box motif. Occupancy of the immediate 5′ upstream SBEs with SMAD2, 3, and 4 and complex formation with p53 are required for TGF-β1-induced PAI-1 expression. USF2 redirects DNA minor grove orientation, promoting interactions between tetramerized p53 bound to its half-site motifs, with SMAD2/3 tethered to the PE2-region SBEs. This conformation facilitates interactions between the MH1 N-terminal domain of SMAD2 and the pN-terminal transactivation domain of p53 and the C terminus of p53 and the MH2 region of SMAD3. p53^S15^ phosphorylation recruits the histone acetylase p300. p53 acetylation and formation of p53/p300 complexes is a rapid response to TGF-β1 and correlates with the time frame of PAI-1 transcriptional activation. The Hippo pathway transcriptional effectors YAP and TAZ interact with SMAD2 and 3 in response to TGF-β1, whereas the AP-1 transcription factor appears to form a complex with YAP-TAZ-TEAD [90] (for references).

**Figure 4:**
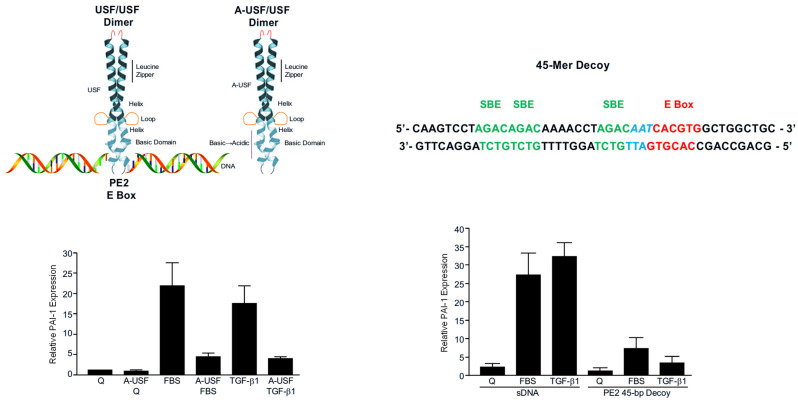
Strategies to interfere with occupancy of the CACGTG motif in the PAI-1 promoter with wild-type USF2 in response to stimulation of G_0_ HaCaT cells with serum or TGF-β1. Keratinocytes were transfected with the dominant-negative A-USF construct which provides a dimer partner for wild-type endogenous USFs but inhibits DNA E box occupancy of the A-USF/USF “heterodimer” due to replacement of the DNA recognition basic domain with acidic residues (**upper left panel**). Cells were serum-starved to initiate quiescence (Q), then stimulated with FBS or TGF-β, for 5 h prior to cell extraction for Western analysis of PAI-1 (**bottom left panel**). Compared to cells not transfected with A-USF constructs, serum-/TGF-β1-induced PAI-1 expression in A-USF transfectants was significantly attenuated (by approximately 80%). HaCaT cells were transfected with 45-mer double-stranded oligonucleotides (**upper right panel**) representing the PE2 E box and the 3 SBEs or sheared control DNA (sDNA), maintained under quiescent conditions then stimulated with FBS or TGF-β1 prior to cell extraction and Western analysis for PAI-1 (**bottom right panel**) [85]. Graphed data illustrate the mean + SD of three independent experiments.

Expression control by USF family members is distinct from simple site recognition, since positive or negative transcriptional outcomes likely depend on cell type, species, and adjoining motifs bound by non-USF factors [5,86,87]. The USF1→USF2 transition at the PE2 E box appears critical to the formation of the higher order transcriptional complex on the PE2 platform (Figure 3). Mobility shift assessments reinforced the finding (with regard to the PAI-1 promoter) that constructs with an intact consensus CACGTG sequence were effective competitors for USF binding while those lacking the CACGTG motif or containing the transcription-attenuating CG → AT mutation failed to inhibit complex formation with a wild-type probe [5,84]. Based on these results, it was important to determine if disruption of USF function would specifically affect PAI-1 transcription in response to serum or TGF-β1. Two approaches were selected to evaluate this possibility. Initially, keratinocytes were engineered to express a dominant-negative A-USF construct in which the wild-type DNA-binding domain was replaced with acidic residues; this modification greatly stabilizes heterodimers formed between A-USF and wild-type USFs and inhibits formation of endogenous USF/DNA complexes [5,6,13,85,88,89]. A-USF expression significantly attenuated both serum- and TGF-β1-induced PAI-1 levels. Secondly, a double-stranded 45 bp PE2 DNA construct was designed based on the previously identified requirements for an intact CACGTG motif for probe recognition by USF [5,84,85]. Similarly to the results obtained with A-USF, transfection of these double-stranded USF-binding “decoys” effectively reduced both serum- and TGF-β1-induced PAI-1 transcript levels in HaCaT keratinocytes [85] (Figure 4).

USF1/2 regulate the transcription of genes that impact cell growth and the proliferative program in various tumor cell types [3]. To address this relationship more specifically, a molecular genetic approach was taken involving transfection of a wild-type USF2 construct in dual p53 mutant HaCaT cells; USF2 overexpression significantly reduced colony formation [85]. To more specifically implicate USF in HaCaT cell proliferative control, a DOX-dependent A-USF expression system was developed to express an HA-tagged dominant-negative A-USF insert (DN-USF) in an inducible Tet-OFF system. Oxytetracycline removal and subsequent A-USF induction resulted in a marked increase in HaCaT cell growth and expression of the validated proliferative biomarker Ki-67 while suppressing both PAI-1 and PAI-2 mRNA levels [85]. Adenoviral-mediated PAI-1 overexpression, moreover, dramatically inhibited keratinocyte proliferation and colony expansion, mimicking the results of USF2 transfection. Collectively, these data demonstrate that the bHLH-LZ transcription factor USF2, and its target gene PAI-1, regulate keratinocyte proliferation.

## 5. Conclusions

Several members of the SERPIN gene family are implicated in tumor progression (SERPINE1, SERPINE2, SERPINE3, SERPINB9, SERPINI1), although SERPINE1 (PAI-1) is the SERPIN most prominently and causatively involved in the creation of a highly aggressive malignant phenotype [91,92,93,94]. Indeed, as previously detailed, PAI-1 regulates critical pathophysiological events in the TME, including angiogenesis and metastasis, immunosuppression (by recruiting M2-polarized macrophages and inducing PD-L1 expression), stromal remodeling, and resistance to chemotherapy (via and glycolytic reprogramming [36,37,46,48,73,76,77]. While high tumor levels are clearly associated with poor outcomes, in other cell types PAI-1 promotes a senescence-like growth arrest [54,83] which contributes to what is known as the PAI-1 “paradox” in cancer [95]. This review intended to provide a comprehensive overview of these developments, at least with regard to the tumor-promoting SERPINE1 gene, and the role of USF2 in SERPINE1 transcriptional control with a focus on various strategies that might have applicability as transcription-targetable agents.

Changes in transcriptional outputs is a hallmark of cancer and a convergence point of oncogenic signaling. Transformed cells often develop a dependency on such genomic reprogramming, highlighting the therapeutic potential of rectifying cancer-associated transcriptional abnormalities in malignant cells. Transcription therapy is an emerging strategy that intends to rectify aberrant gene expression in cancer cells through direct intervention in the transcription process. The transcriptional apparatus was previously considered undruggable due, in large measure, to the perception that (a) transcription is a nuclear event and therefore not readily accessible to therapeutic agents, and (b) many transcriptional components lack enzymatic activity and, thus, are unsuitable for chemically directed interventions [96]. In recent years, approaches and “therapeutic” agents, including transcription factor decoys and dominant-negative constructs, have been developed that target various levels of transcriptional regulation [96]. It has been estimated that at least 10% of FDA-approved anticancer drugs that were not initially developed to target transcription actually regulate this process in one way or another [96]. In recent years, however, great progress has been made in efforts to develop transcription-targeted therapeutic agents. Some of these agents are approved for clinical practice or have entered clinical trials for cancer treatments. Double-strand oligodeoxynucleotides (ODNs) or hairpin single strand ODNs are particularly effective decoys. These ODNs are designed to encompass recognition sequences for specific transcription factors or contain modified sequences that provide for significantly increased protein–DNA interactions [96]. Transfected decoys bind corresponding transcription factors, effectively sequestering them away from their target promoters, thereby regulating gene expression. Indeed, decoys targeting transcription factors including Sp1, AP-1, STAT3, and Ets-1 inhibit expression of cancer associated genes (e.g., VEGF, uPAR, and Bcl-XL) and attenuate growth and metastasis of various cancer cells [96]. Recent advances in design and fabrication have demonstrated the feasibility of developing decoys capable of discriminating among closely related transcription factors. However, as is true for other oligonucleotide-based “chemotherapeutic” agents, the delivery of transcription factor decoys to target cells in vivo and in vivo stability are two major challenges before clinical applications become a reality. As one example of a potential druggable target in a tumor progression gene, this review highlights the role of the USF1→USF2 transition in SERPINE1 transcription. Since PAI-1 is a central regulator of tumor progression and therapeutic resistance in cutaneous malignancies [94,95], this review focused on highlighting the consequences of disrupting the USF2/PAI-1 transcription network in p53 mutant human keratinocytes.

## Data Availability

No new data were created or analyzed in this study.

## Abbreviations

bHLH-LZ, basic helix-loop-helix/leucine zipper; USF, upstream stimulatory factor; CDK2, cyclin-dependent kinase 2; CDK5, cyclin-dependent kinase 5; CK2, casein kinase 2; DNA-PK, DNA-dependent protein kinase; GSK3β, glycogen synthase kinase 3 beta; CHCHD4, coiled-coil-helix-coiled-coil-helix domain containing 4 protein; lncRNA-NEAT1, long non-coding RNA-nuclear paraspeckle assembly transcript 1; TF-ETV5, ETS variant transcription factor 5; ZNF641, zinc finger protein 641; SEZ6L2. Seizure related 6 homolog like protein 2; TUG1, taurine-upregulated gene 1; EMT, epithelial-to-mesenchymal transition; SLC25A17, solute carrier family 25 member 17; GFP, green fluorescent protein; HNSCC, head and neck squamous cell carcinoma; CAF, cancer-associated fibroblasts; SMA, smooth muscle alpha actin,; BZW2, basic leucine zipper and W2 domain-containing protein 2; LAMP3, lysosome-associated membrane protein 3; TGF-β1, transforming growth factor-β1; SERPINE1, serine protease inhibitor-1; CUB, CUB domain containing protein; Coll, collagen;; FN, fibronectin; uPA, urokinase plasminogen activator; MMP,; matrix metalloproteinase; TME, tumor microenvironment; uPA, urokinase plasminogen activator; LRP1, low-density lipoprotein receptor-related protein1; uPAR, urokinase plasminogen activator receptor; PAI-1, plasminogen activator inhibitor-1.
